# Opinions and Knowledge of Midwives Regarding Physical Exercise During Pregnancy: Insights and Implications for Midwifery Practice

**DOI:** 10.7759/cureus.71318

**Published:** 2024-10-12

**Authors:** Athina Diamanti, Maria Christina Koutsogianni, Maria Iliadou, Vasiliki E Georgakopoulou, Victoria Vivilaki

**Affiliations:** 1 Department of Midwifery, University of West Attica, Athens, GRC; 2 Department of Pathophysiology/Pulmonology, Laiko General Hospital, Athens, GRC

**Keywords:** health promotion, maternal health, midwives, physical exercise, pregnancy

## Abstract

Background

Physical exercise during pregnancy offers significant benefits for maternal and fetal health, improving cardiovascular fitness, managing weight, and reducing risks of gestational diabetes and hypertension. Despite these benefits, the role of midwives in promoting exercise is critical but often underexplored.

Materials and methods

This cross-sectional quantitative study surveyed 172 midwives using a structured questionnaire to assess their knowledge and attitudes towards physical exercise during pregnancy. Descriptive and inferential statistics, including chi-square tests, were used to analyze the data. Data collected through the questionnaires were analyzed using IBM SPSS Statistics v. 29 (IBM Corp., Armonk, NY).

Results

Among the 172 participants, 97.7% were female with a median age of 36 years. While 65.1% engaged in physical exercise themselves, only 26.7% recommended exercise during pregnancy to their patients. The knowledge level of midwives about the benefits of exercise was high, with 95.3% recognizing its role in maintaining hormonal balance and 88.4% understanding its benefits in reducing gestational diabetes. However, 32.6% of midwives reported not feeling confident in guiding pregnant women on how to exercise. Statistically significant correlations were found between midwives' opinion level and their knowledge of exercise benefits, including proper weight gain (rho = 0.263, p = 0.01) and reduced likelihood of gestational diabetes (rho = 0.492, p = 0.01).

Conclusions

Midwives recognize the importance of exercise during pregnancy but require more training to provide effective guidance. Enhancing midwives' education on exercise can improve maternal and fetal health outcomes by promoting physical activity during pregnancy.

## Introduction

Physical exercise during pregnancy is a topic of increasing interest due to its significant benefits for both maternal and fetal health [1]. Regular physical activity during pregnancy is essential for enhancing cardiovascular fitness, managing weight gain, reducing the risk of gestational diabetes and hypertension, and improving psychological well-being. For instance, women who engage in regular moderate-intensity exercise during pregnancy show a 30% improvement in cardiovascular fitness and are less likely to experience excessive weight gain compared to those who do not exercise [1]. These benefits are crucial during a period marked by profound physiological changes.

Physical exercise also helps improve the respiratory, cardiovascular, and musculoskeletal systems, aiding women in adapting to these changes. Moreover, exercise can reduce common pregnancy-related issues such as back pain, swelling, and fatigue. Studies have shown that pregnant women who exercise report a 20-40% reduction in lower back pain [2]. Physical activity also improves posture, muscle tone, and gastrointestinal function. Additionally, it can decrease the duration of labor by up to 90 minutes and lower the likelihood of cesarean sections by 25%, contributing to better birth outcomes [3].

From a psychological perspective, exercise is known to release endorphins, which can alleviate stress and anxiety, common issues faced by pregnant women. Improved mood and energy levels resulting from regular physical activity contribute to a better pregnancy experience and reduce the likelihood of postpartum depression [4]. For the fetus, maternal physical activity is associated with numerous benefits as well. Babies born to physically active mothers have a 10% lower risk of being born macrosomic, which helps prevent birth complications. Furthermore, maternal exercise is linked to a 30% reduction in the risk of childhood obesity and better neurodevelopmental outcomes [5].

The role of midwives in promoting and guiding physical exercise during pregnancy is pivotal. Midwives are often the primary source of advice for pregnant women, making their knowledge and attitudes towards exercise essential for effective health promotion [6]. While midwives generally recognize the benefits of exercise during pregnancy, there are gaps in their knowledge, particularly regarding the specifics of exercise regimens and their impact on childbirth outcomes [7].

This study adopts a quantitative research approach using a structured questionnaire to assess the knowledge and attitudes of midwives towards exercise during pregnancy. By examining the responses from a diverse group of healthcare professionals, predominantly midwives, this research aims to identify gaps in knowledge and areas where further training and support may be needed.

## Materials and methods

Research design

This is a cross-sectional study that utilized a quantitative research approach to investigate the opinions and knowledge of midwives regarding physical exercise during pregnancy. A structured questionnaire was employed as the primary data collection tool, administered through convenience sampling. The design aimed to capture both descriptive and inferential data to understand better the current practices and beliefs among healthcare professionals in this field​​​​.

Participants

The study population consisted of midwives working in various healthcare settings, including public hospitals, private practices, and clinics in the greater Athens area and rural regions.

Data collection tool

A structured questionnaire was developed and validated for data collection. The questionnaire comprised 55 items, including 51 closed-ended questions and 4 open-ended questions. The questions were divided into four main sections: demographic information, knowledge about physical exercise during pregnancy, attitudes towards physical exercise during pregnancy, and professional practices regarding exercise recommendations (Appendix)​​​​.

Sampling procedure

The sampling method employed was convenience sampling, selected due to the accessibility of participants within the researcher's professional network, predominantly linked to Elena Venizelou Hospital in Athens. The researcher's professional network comprised midwives working in this hospital as well as affiliated healthcare professionals from nearby clinics and maternity centers. These midwives had varying levels of experience and expertise, ranging from recent graduates to those with decades of clinical practice in both urban and rural healthcare settings. 

Statistical analysis

Reliability analysis of the questionnaire was performed using Cronbach's alpha, while validity was assessed through factor analysis techniques including the Kaiser-Meyer-Olkin measure and Bartlett's test of sphericity​​. Normality of the data was tested using Kolmogorov-Smirnov and Shapiro-Wilk tests to determine the appropriate statistical tests for hypothesis testing. Means, standard deviations, and frequencies were used to summarize the demographic data and responses to the questionnaire items. All continuous variables had no normal distribution and therefore, all inferential tests were performed using non-parametric indicators. Mann-Whitney U test and Spearman's Rank Correlation Coefficient were used for hypotheses testing due to the non-normal distribution of data. The level of statistical significance was set at 0.05. Data collected through the questionnaires were analyzed using IBM SPSS Statistics 29 (IBM Corp., Armonk, NY).

Ethical considerations

Ethical approval for the study was obtained from the Institutional Review Board of Elena Venizelou Hospital (protocol number 6912/15-12-22). Participants provided informed consent, ensuring the confidentiality and anonymity of their responses. The data collected were used exclusively for research purposes and were securely stored and destroyed after the completion of the study​​.

## Results

Demographic characteristics of the study population and data on physical exercise during pregnancy

The questionnaire exhibited strong reliability and validity. Reliability was confirmed with a Cronbach’s alpha of 0.813, while validity was supported by the Kaiser-Meyer-Olkin (KMO) measure of sampling adequacy (0.821) and Bartlett’s test of sphericity (p < 0.0001), indicating that the data were appropriate for factor analysis.

A total of 172 midwives participated in the study, with the vast majority being female (97.7%, n = 168) and only a small proportion male (2.3%, n = 4). Participants' ages ranged from 22 to 60 years, with a median age of 36. All respondents were of Greek nationality.

Educational attainment among the participants was high, with the vast majority being graduates (98.8%, n = 170), while only 1.2% (n = 2) were final-year midwifery students. Notably, 43% (n = 74) of the midwives had pursued advanced education, earning master’s degrees, and 2.3% (n = 4) had obtained a doctorate. 

In terms of employment, nearly half of the participants (47.7%, n = 82) were employed in public institutions, while 37.2% (n = 64) worked in the private sector. A smaller proportion were self-employed (9.3%, n = 16), and 5.8% (n = 10) were unemployed. The median length of work experience was 10 years, with a range spanning from less than a year to 36 years.

Regarding family status, 66.3% (n = 114) of the participants had children, while 33.7% (n = 58) did not. A majority (65.1%, n = 112) reported participating in some form of physical exercise, whereas 34.9% (n = 60) did not engage in any exercise. Of those who exercised, more than half (50.6%, n = 87) spent 1-2 hours per week on physical activity, and 14.5% (n = 25) exercised for 2-4 hours weekly. However, only 26.7% (n = 46) reported continuing to exercise during pregnancy. 

In terms of preferred physical activities, Pilates was the most popular choice, favored by 47.4% (n = 74) of participants. Other preferred activities included swimming (15.4%, n = 24) and yoga (10.3%, n = 16). Additionally, 26.9% (n = 42) engaged in other forms of exercise, such as Crossfit (2.3%, n = 4), total body resistance exercise (TRX) (1.2%, n = 2), weight training (1.2%, n = 2), volleyball (1.2%, n = 2), gym workouts (1.2%, n = 2), high-intensity interval training (HIIT) (1.2%, n = 2), cycling (1.2%, n = 2), aerobic exercise (2.3%, n = 4), walking (8.1%, n = 14), and running (4.7%, n = 8). Detailed demographic characteristics and data on exercise habits during pregnancy are presented in Table 1.

**Table 1:** Demographic characteristics of the study population and data on physical exercise during pregnancy. The data is represented as N (%) and as median (range). Aside from the age and years of service, all values are expressed in frequencies. HITT: high-intensity interval training; TRX: total (body) resistance exercise

Parameters	Values	Percentage (%)
Age in years; median (range)	36 (22-60)	-
Years of service; median (range)	10 (0-36)	-
Gender		
Females	168	97.7
Males	4	2.3
Level of education	-	-
Midwifery student	2	1.2
Midwifery graduate	170	98.8
Further education	-	-
Master (MSc)	74	43
Doctorate (PhD)	4	2.3
Employment at midwifery institution	-	-
Public employee	82	47.7
Private employee	64	37.2
Self-employed	16	9.3
Unemployed	10	5.8
Children	-	-
Yes	114	66.3
No	58	33.7
Physical exercise	-	-
Yes	112	65.1
No	60	34.9
Duration of physical exercise (hours per week)	-	-
None	60	34.9
1-2 hours	87	50.6
2-4 hours	25	14.5
Exercise during pregnancy	-	-
Yes	46	26.7
No	62	36
Reasons for not exercising during pregnancy	-	-
None	46	74.2
Lack of desire	8	12.9
Bedrest	2	3.2
Contractions	2	3.2
Fear	4	6.5
Preferred forms of physical exercise	-	-
Pilates	74	47.4
Yoga	16	10.3
Swimming	24	15.4
Other	42	26.9
Other forms of physical exercise	-	-
None	130	75.6
Crossfit	4	2.3
TRX	2	1.2
Aerobic	4	2.3
Weights	2	1.2
Volleyball	2	1.2
Gym	2	1.2
HIIT	2	1.2
Walking	14	8.1
Cycling	2	1.2
Running	8	4.7

Knowledge about physical exercise during pregnancy

The knowledge levels of midwives regarding physical exercise during pregnancy, Kegel exercises, the impact of exercise on childbirth, and exercise during the postpartum period are detailed in Table 2.

**Table 2:** The knowledge levels of midwives regarding physical exercise during pregnancy, Kegel exercises, the impact of exercise on childbirth, and exercise during the postpartum period. The data is represented as N (%)

Question	N	%
Knowledge of Midwives About Exercise During Pregnancy		
All women can exercise during pregnancy		
True	36	20.9
False	136	79.1
Exercise during pregnancy contributes to proper weight gain		
True	164	95.3
False	8	4.7
A woman who did not exercise before pregnancy can start during pregnancy		
True	118	68.6
False	54	31.4
An obese woman can exercise during pregnancy		
True	166	96.5
False	6	3.5
Exercise helps prevent obesity after childbirth		
True	162	94.2
False	10	5.8
Exercise helps maintain hormonal balance in pregnant women		
True	164	95.3
False	8	4.7
Exercise reduces anxiety and stress during pregnancy		
True	168	97.7
False	4	2.3
Exercise helps pregnant women accept body changes more easily		
True	158	91.9
False	14	8.1
Exercise helps prevent depression before and after childbirth		
True	168	97.7
False	4	2.3
Exercise reduces the likelihood of developing gestational diabetes		
True	152	88.4
False	20	11.6
Exercise reduces swelling and back pain		
True	150	87.2
False	22	12.8
Knowledge of Midwives About Kegel Exercises		
Kegel exercises should be done during pregnancy		
True	156	90.7
False	16	9.3
Kegel exercises prepare pelvic floor muscles for birth		
True	160	93
False	12	7
Kegel exercises should be done during postpartum		
True	154	89.5
False	18	10.5
Knowledge of Midwives About the Impact of Exercise on Childbirth		
Exercise reduces the likelihood of preeclampsia		
True	110	64
False	62	36
Exercise does not help reduce anxiety during childbirth		
True	156	9.3
False	16	90.7
Exercise during pregnancy does not reduce the average time of labor		
True	48	27.9
False	124	72.1
Exercise does not reduce the use of pain management medications		
True	44	25.6
False	128	74.4
Knowledge of Midwives About Exercise During the Postpartum Period		
Faster recovery of abdominal muscles after childbirth		
True	164	95.3
False	8	4.7
A breastfeeding woman should not exercise		
True	20	11.6
False	152	88.4

A large majority of midwives demonstrated a strong understanding of the benefits of exercise during pregnancy. For example, 96.5% (n = 166) recognized that physical activity supports proper weight gain, and 93% (n = 160) were aware that obese women can safely exercise during pregnancy. Furthermore, 97.7% (n = 168) acknowledged that exercise helps reduce depression both before and after childbirth.

Knowledge of Kegel exercises was similarly high, with 93% (n = 160) understanding their role in preparing the pelvic floor muscles for birth. However, awareness was somewhat lower regarding exercise’s impact on specific childbirth outcomes, as only 64% (n = 110) identified that exercise reduces the risk of preeclampsia. On the other hand, understanding of the benefits of postpartum exercise was strong, with 95.3% (n = 164) recognizing its role in accelerating the recovery of abdominal muscles.

The opinions of midwives towards exercise during pregnancy and their professional roles in guiding exercise methods

The opinions of midwives towards exercise during pregnancy and their professional roles in guiding exercise methods are displayed in Table 3.

**Table 3:** Opinions of Midwives Regarding Exercise During Pregnancy and Their Professional Role in Guiding Exercise The data is represented as N (%).

Questions	N	%
Opinions of Midwives Towards Exercise During Pregnancy		
Women with a normal pregnancy should exercise		
Agree	166	96.5
Disagree	6	3.5
Only a fitness instructor can undertake pregnancy exercises		
Agree	34	19.8
Disagree	138	80.2
I know how to guide a pregnant woman on how to exercise during pregnancy		
Agree	116	67.4
Disagree	56	32.6
I know how to guide a postpartum woman on how to exercise during the postpartum period		
Agree	116	67.4
Disagree	56	32.6
Exercise is not the midwife's concern; other issues take priority		
Agree	58	33.7
Disagree	114	66.3
A breastfeeding woman should not exercise		
Agree	4	2.3
Disagree	168	97.7
An overweight woman should not exercise during pregnancy		
Agree	12	7
Disagree	160	93
Pregnant women should be educated on the exercises they are allowed to do		
Agree	164	95.3
Disagree	8	4.7
Midwives should inform pregnant women about exercise methods		
Agree	168	97.7
Disagree	4	2.3
Pregnant women should not exercise before six months		
Agree	38	22.1
Disagree	134	77.9
Opinions of Midwives on Exercise Methods During Pregnancy		
Swimming is the best exercise method during pregnancy		
Agree	132	76.7
Disagree	40	23.3
Yoga is the best exercise method during pregnancy		
Agree	142	82.6
Disagree	30	17.4
Pilates is the best exercise method during pregnancy		
Agree	146	84.9
Disagree	26	15.1
Walking is the best exercise method during pregnancy		
Agree	150	87.2
Disagree	22	12.8
Aerobic exercise is the best exercise method during pregnancy		
Agree	158	91.9
Disagree	14	8.1
Pregnant women should do perineal exercises		
Agree	160	93
Disagree	12	7
Pregnant women should do abdominal exercises		
Agree	84	48.8
Disagree	88	51.2
Pregnant women should do leg exercises		
Agree	168	97.7
Disagree	4	2.3

The majority of midwives (96.5%, n = 166) agreed that women with a normal pregnancy should engage in physical activity, and an even higher proportion (97.7%, n = 168) believed that pregnant women should be educated about the exercises they are allowed to perform. Furthermore, 93% (n = 160) of midwives disagreed with the notion that overweight women should refrain from exercising during pregnancy. Despite this strong agreement on the value of exercise, only 67.4% (n = 116) felt confident in providing guidance to pregnant and postpartum women on appropriate exercise routines, highlighting a significant area for professional development.

Regarding the best forms of exercise for pregnant women, walking was the most favored, supported by 87.2% (n = 150) of midwives, while 91.9% (n = 158) endorsed aerobic exercises. Yoga and Pilates were also well-regarded, with 82.6% (n = 142) and 84.9% (n = 146) of midwives recommending them, respectively. However, opinions were divided when it came to abdominal exercises during pregnancy, with responses nearly split down the middle: 48.8% (n = 84) in favor and 51.2% (n = 88) opposed.

Testing Research Hypotheses

Research Hypothesis 1: Is the knowledge level of midwives regarding physical exercise during pregnancy different between those who exercise and those who do not?

Before testing this hypothesis, a new variable was created to define the knowledge level of midwives about exercise during pregnancy. The results show a mean knowledge level of 1.279 with a standard deviation of 0.1106, a maximum value of 1.600, and a minimum value of 1.00, based on a range of dichotomous responses (1.00 to 2.00).

The mean knowledge level for midwives who exercised was 1.275 ± 0.1026, while for those who did not exercise, it was 1.286 ± 0.1248. There were no statistically significant differences between these two groups in terms of their knowledge level regarding physical exercise during pregnancy (p = 0.668).

Research Hypothesis 2: Is there a correlation between whether midwives exercise and the type of exercise they choose with their knowledge level about physical exercise during pregnancy?

No statistically significant correlations were found between the fact that midwives exercise and the type of exercise they prefer with their knowledge level about physical exercise during pregnancy (rho = 0.033, p = 0.669; rho = 0.066, p = 0.411).

Research Hypothesis 3: Is there a correlation between the experience of midwives and their knowledge level about physical exercise during pregnancy?

The results indicate no statistically significant correlation between the years of experience of midwives and their knowledge level regarding physical exercise during pregnancy (rho = -0.111, p = 0.146).

Research Hypothesis 4: Is there a correlation between midwives' knowledge level and their opinion that women should exercise during pregnancy?

The results show no statistically significant correlation between the midwives' knowledge level and their opinion on whether women should exercise during pregnancy (rho = 0.126, p = 0.100).

Research Hypothesis 5: Is there a correlation between midwives' knowledge level about physical exercise during pregnancy and their opinions on (a) women exercising during pregnancy, (b) women being educated on exercise, and (c) women choosing the best exercise method?

There was no statistically significant correlation between midwives' knowledge level and their opinion on whether pregnant women should be educated about exercises they are allowed to perform (rho = -0.026, p = 0.732). However, statistically significant correlations were found between midwives' knowledge level and their opinions on whether women should exercise during pregnancy and on choosing the best exercise method (rho = 0.408, p = 0.001 and rho = 0.171, p = 0.025, respectively).

Research Hypothesis 6: Is there a correlation between midwives' knowledge of physical exercise during pregnancy and its associated benefits?

This hypothesis explores the relationship between midwives' knowledge of physical exercise during pregnancy and the perceived benefits of such activities. The analysis demonstrated statistically significant correlations between the midwives' knowledge levels and their perspectives on various benefits of prenatal exercise, as outlined in Table 4.

**Table 4:** Correlations between midwives' knowledge level and their opinion on several benefits of exercise during pregnancy. The value at which the p-value was considered significant was p<0.05. Spearman's correlation coefficient test was used for the statistical analysis.

Knowledge	Spearman’s correlation coefficient	p-value
Women should exercise during pregnancy	0.126	0.1
Exercise contributes to proper weight gain	0.263	0.001
Exercise helps maintain hormonal balance	0.095	0.216
Exercise reduces anxiety and stress during pregnancy	0.022	0.771
Exercise helps accept body changes	0.245	0.001
Exercise prevents depression before and after childbirth	0.241	0.001
Exercise reduces the likelihood of preeclampsia	0.573	0.001
Exercise reduces the likelihood of gestational diabetes	0.492	0.001
Exercise reduces swelling and back pain	0.53	0.001
Exercise does not reduce average labor time	0.407	0.001
Exercise does not reduce pain management medication use	0.187	0.014
Faster recovery of abdominal muscles after childbirth	0.04	0.602

A moderate positive correlation was observed between midwives' opinions and their awareness that exercise contributes to appropriate weight management during pregnancy (rho = 0.263, p = 0.001). Midwives with more favorable opinions were also more likely to acknowledge that physical exercise eases the acceptance of bodily changes during pregnancy (rho = 0.245, p < 0.001). Additionally, there was a significant link between the opinions of midwives and knowing that exercise can help prevent depression before and after childbirth (rho = 0.241, p = 0.001).

The strongest correlation was between the midwives' opinions and the understanding that exercise decreases the risk of preeclampsia (rho = 0.573, p < 0.001). Higher opinion levels also corresponded with greater awareness of exercise's role in reducing the likelihood of gestational diabetes (rho = 0.492, p < 0.001), and a substantial positive correlation was noted between opinion levels and the knowledge that exercise alleviates swelling and back pain (rho = 0.531, p < 0.001).

Furthermore, midwives with more positive opinions were more likely to be aware that exercise can shorten the average duration of labor (rho = 0.407, p < 0.001). Lastly, a weaker yet significant correlation was found between midwives' opinions and the knowledge that exercise reduces the need for pain management medications during labor (rho = 0.187, p = 0.014).

## Discussion

Physical exercise during pregnancy is widely recognized for its numerous benefits, including improved cardiovascular fitness, proper weight management, and reduced risks of gestational diabetes and hypertension [1-3]. Regular exercise also enhances psychological well-being, reducing stress and anxiety [4]. Despite these well-documented advantages for both maternal and fetal health, midwives play a pivotal role in promoting and guiding physical activity during pregnancy. However, knowledge gaps remain regarding the specifics of exercise regimens and their impact on pregnancy and childbirth outcomes. This study aimed to explore the knowledge and attitudes of midwives towards physical exercise during pregnancy, highlighting areas where further education and support may be necessary to optimize maternal care.

The main findings of this study reveal that 172 professional midwives participated, demonstrating a high level of knowledge about exercise during pregnancy. The participants highlighted the importance of physical activity for women during pregnancy and the postpartum period without complications, recommending appropriate types of exercise such as swimming, yoga, pilates, walking, aerobic exercises, perineal exercises, abdominal exercises, and lower limb exercises, with yoga being the most preferred. They also emphasized the necessity for proper guidance from healthcare professionals for women facing special conditions such as obesity, gestational diabetes, or those who are breastfeeding. Furthermore, the study underscores the importance of continuous professional development for midwives to familiarize themselves with exercise-related issues during pregnancy and the postpartum period, as well as their professional rights.

Studies like those by Kime et al. [8] and Crampton et al [9] have shown that pregnant women who received advice on physical exercise from midwives during the prenatal period achieved higher levels of physical activity compared to those who did not receive such advice​. Consequently, it is the responsibility of midwives to provide correct, safe, and achievable guidelines for physical activity during pregnancy and the postpartum period. The literature identified a knowledge gap among midwives regarding physical activity and the limitations of providing exercise advice to pregnant and postpartum women. Midwives acknowledged the importance of physical activity in enhancing maternal health outcomes and expressed their readiness to deliver effective education and physical activity counseling. However, they emphasized the need for empowerment through training and workshops on the benefits of exercise, suitable types of exercise for women at each stage of pregnancy, and the risks and precautions for those with complicated obstetric conditions. Researchers suggest that midwives should receive more training to provide better prenatal care, and their guidance should play a significant role in promoting an active lifestyle among pregnant and postpartum women. Following an educational intervention, healthcare professionals would see an improvement in their knowledge and practices regarding physical activity during pregnancy and the postpartum period​ [8, 9].

In their study involving a sample of 16 women during pregnancy and postpartum, Findley et al. [10] identified reasons why physical activity tends to decrease during pregnancy and remains low in the early postpartum period, despite its known physical and psychological benefits. Women's experiences revealed attitudes toward physical exercise during pregnancy and postpartum, as well as the decision-making processes related to physical exercise during these periods. The data analysis identified reasons such as poor body image, a lack of emphasis on maintaining physical fitness during pregnancy and motherhood, expectations from physical exercise, pressure to comply with a specific exercise regimen, concerns about potential exercise complications, and unclear advice from the midwife. Decision-making regarding physical exercise during pregnancy and postpartum was influenced by pressure from family, friends, and the social environment, who felt responsible for protecting the woman from harm; psychological pressure from social media for weight loss immediately after childbirth; women's beliefs about the benefits of maintaining physical fitness; and their expectations about how active they thought they could be during pregnancy. Women viewed pregnancy as "uncharted territory," expressing fear about their feelings toward their bodies, and often found midwives' advice lacking or not tailored to everyone. These findings align with the present study's findings, which highlighted the lack of desire and inability to exercise, as well as the fear that makes decision-making regarding physical activity during pregnancy and postpartum difficult. To support women in making informed decisions about their exercise behavior, researchers recommend developing detailed and personalized advice as part of obstetric care, considering the physical and psychological aspects of physical exercise during pregnancy [10].

Another study by Okafor and Goon [11] focused on providing education and physical activity counseling during pregnancy with a sample of 17 midwives. Midwives rarely provided education and advice on physical exercise to pregnant women, citing a lack of knowledge about which physical activity to recommend, the duration, and the intensity of the activity. Walking was the only physical activity unequivocally recommended during prenatal sessions. However, they expressed concerns about jogging, swimming, and gym activities being unsafe for a pregnant woman and the fetus. Barriers to providing education and counseling on physical activity included a shortage of midwives, high workload responsibilities, the unavailability of exercise equipment, and a lack of prioritization of prenatal physical activity. Midwives also reported professional responsibilities resulting in excessive fatigue, making it difficult for them to advise women. They supported the introduction of exercise courses in prenatal healthcare and training midwives in exercises to enhance their knowledge about prenatal exercise and their engagement with pregnant and postpartum women​ [11]​. Both the study by Okafor and Goon and the current study reveal significant gaps in midwives' ability to confidently provide physical activity counseling during pregnancy. In Okafor and Goon’s study, midwives were primarily comfortable recommending walking, while expressing concerns about activities like swimming or jogging [11]. In contrast, our study indicated that while midwives acknowledged a broader acceptance of various exercises, including Pilates, swimming, and yoga, active recommendation of these activities to patients was limited, suggesting persistent uncertainty despite recognizing their benefits. Both studies identified barriers such as heavy workloads and lack of training as key reasons midwives were hesitant to provide exercise advice. While midwives in our study had higher theoretical knowledge, with 95.3% understanding the benefits of exercise for hormonal balance, they, like those in Okafor and Goon’s study, lacked confidence in translating that knowledge into practical advice. Both studies emphasize the need for enhanced training and support to empower midwives to promote physical activity more effectively during pregnancy.

Another study by Hayman et al. [12] focused on exercise during pregnancy, which is associated with various health benefits for both mother and child. Despite these benefits, only a few pregnant women in the study were sufficiently active in physical exercise during pregnancy. Midwives can play a crucial role in encouraging women to be active during pregnancy and postpartum by providing clear and accurate exercise advice. A study involving 113 women revealed that 53% of them reported receiving some form of exercise advice. Specifically, 34% of women discussed the frequency of exercise, 57% discussed the intensity of exercise, and 39% discussed the duration of exercise. Healthcare professionals may not actively provide exercise advice to pregnant women. Researchers suggest that healthcare professionals provide exercise advice to pregnant and postpartum women, and if they lack the necessary knowledge, they should improve it through education and support to provide personalized and reliable exercise advice​ [12]​.

Healthcare professionals in midwifery play a critical role in educating pregnant and postpartum women about an active lifestyle. Researchers could conduct further research using a quantitative and qualitative approach to explore the benefits of physical exercise during and after pregnancy. They could also explore potential correlations between samples from various healthcare institutions, potentially leading to cross-checks and comparative studies [13]. To empower healthcare professionals to promote physical activity during the perinatal period, researchers should conduct research on a larger sample of midwives, obstetricians, doctors, and women, highlighting their attitudes and drawing conclusions from them​.

One of the key strengths of this study is its comprehensive approach to assessing both the knowledge and attitudes of midwives toward physical exercise during pregnancy, offering valuable insights into areas for improvement. The study also involved a relatively large sample size of 172 midwives, providing a robust dataset that allows for generalizable conclusions within the context of Greek healthcare settings. Another strength is the use of a structured questionnaire validated for reliability and validity, ensuring that the data collected was consistent and accurately reflected the midwives' knowledge and opinions. Additionally, the study explored a wide range of factors, from exercise recommendations to midwives’ confidence in guiding pregnant women, revealing nuanced correlations such as the statistically significant link between knowledge levels and attitudes toward the benefits of exercise (e.g., rho = 0.263 for proper weight gain, p < 0.01). These findings provide a clear pathway for targeted education and interventions aimed at improving midwives’ roles in promoting maternal and fetal health through physical activity.

The current study has some limitations. The study utilized convenience sampling, which may limit the generalizability of the findings. Participants were selected based on accessibility within the researcher's professional network, potentially introducing selection bias and not fully representing the broader population of midwives. The study was conducted primarily in the greater Athens area and rural regions, which may not reflect the knowledge and attitudes of midwives in other regions or countries. Cultural, educational, and healthcare practice differences could affect the applicability of the results to a wider context. The study relied on self-reported data through structured questionnaires. This approach is subject to social desirability bias, where participants might provide responses, they perceive as favorable rather than their true opinions or behaviors. Additionally, recall bias could affect the accuracy of the reported information. Although the questionnaire demonstrated high reliability and validity, the use of closed-ended questions may have limited the depth of responses. Open-ended questions were minimal, potentially restricting the richness of qualitative insights that could provide a more nuanced understanding of the midwives' knowledge and attitudes.

The study provides a snapshot of the current knowledge and attitudes of midwives but does not capture changes over time. Longitudinal studies could better assess how knowledge and attitudes evolve with ongoing training and professional development. Despite high overall knowledge levels, significant gaps were identified in midwives' confidence in guiding pregnant women on physical exercise. This indicates that knowledge does not necessarily translate into practical application, highlighting the need for more targeted training and support. The high percentage of female participants (97.7%) might not provide a comprehensive view of the opinions and knowledge across all genders within the profession. Furthermore, the study's reliance on responses from midwives with varying levels of experience and education may result in a heterogeneous dataset that complicates the interpretation of results. The study did not extensively control for potential confounding factors such as the specific healthcare settings (public vs. private), personal beliefs about physical exercise, or previous experiences with exercise during pregnancy, which could influence the participants' responses.

## Conclusions

The study reveals that while midwives acknowledge the significant benefits of physical exercise during pregnancy, there are considerable gaps in their confidence and knowledge regarding specific exercise recommendations. Although a substantial proportion of the midwives engage in physical exercise themselves, only a minority actively encourage pregnant women to do the same. This highlights a critical need for targeted education and training programs to empower midwives with the necessary skills and confidence to promote physical activity effectively. By bridging these knowledge gaps, midwives can play a pivotal role in enhancing maternal and fetal health outcomes through informed exercise guidance during pregnancy.

## Appendices

Section A

Table 5 shows the demographic data section of the questionnaire.

**Table 5:** SECTION A. Demographic data

Question
1. Age: ………
2. Gender
o Male
o Female
3. Nationality
o Greek
o Other
3a. If 'Other Nationality', please specify which: …………………………………
4. Education Level
o Midwifery School Student
o Semester of attendance
o Midwifery School Graduate
5. Additional Education
o Master's Degree (MSc)
o Doctorate (PhD)
6. Midwifery Institution of Employment
o Public Employee
o Private Employee
o Freelancer
o Unemployed
7. Total Years of Experience: ……
8. Do you have children?
o Yes
o No
9. Do you exercise?
o Yes
o No
10. How many hours per week do you exercise?
o None
o 1-2 hours
o 2-4 hours
11. Did you continue to exercise during your pregnancy?
o Yes
o No
11a. If not, why? …………………………………………………………………………………
12. What type of exercise do you prefer?
o Pilates
o Yoga
o Swimming
o Other
12a. If 'Other' type of exercise, please specify: ………………………………………………………

Section B

Table 6 shows Section B of the questionnaire exploring Knowledge about physical exercise during pregnancy. 

**Table 6:** Section B. Knowledge about physical exercise during pregnancy

Question	Correct	Incorrect
1. All women can exercise during their pregnancy.	-	-
2. Exercise during pregnancy contributes to proper weight gain.	-	-
3. A woman who did not exercise before can start during pregnancy for the first time.	-	-
4. An obese woman can exercise during her pregnancy.-	-	-
5. Exercise helps prevent obesity after childbirth.-	-	-
6. Exercise helps maintain hormonal balance in pregnant women.-	-	-
7. Exercise reduces anxiety and stress during pregnancy.-	-	-
8. Exercise helps pregnant women accept body changes more easily.-	-	-
9. Exercise helps prevent depression before and after childbirth.-	-	-
10. Exercise reduces the likelihood of preeclampsia.-	-	-
11. Exercise reduces the likelihood of gestational diabetes.-	-	-
12. Exercise reduces swelling and back pain.-	-	-
13. Kegel exercises should be done during pregnancy.-	-	-
14. Kegel exercises properly prepare the pelvic floor muscles for childbirth.-	-	-
15. Kegel exercises should be done postpartum.-	-	-
16. Exercise does not help combat anxiety during childbirth.-	-	-
17. Exercise during pregnancy does not reduce the average labor time for women who exercise.-	-	-
18. Exercise does not reduce the need for pain management medications for women who exercise.-	-	-
19. Abdominal muscles recover faster after childbirth.-	-	-
20. A breastfeeding woman should not exercise.-	-	-

SECTION C

Table 7 shows Section C of the questionnaire exploring the opinions of midwives towards exercise during pregnancy.

**Table 7:** Section C. The opinions of midwives towards exercise during pregnancy

Question	Agree	Disagree
1. Women with a normal pregnancy should exercise.	-	-
2. Only a trainer can undertake exercises during pregnancy.	-	-
3. I know how to guide a pregnant woman to exercise during pregnancy.	-	-
4. I know how to guide a postpartum woman to exercise.	-	-
5. Exercise is not a midwife's concern; other issues take precedence in the midwifery care of pregnant women.	-	-
6. A breastfeeding woman should not exercise.	-	-
7. An overweight woman should not exercise during her pregnancy.	-	-
8. Pregnant women should be trained in the exercises allowed during pregnancy.	-	-
9. Midwives should know and inform pregnant women whether they can exercise, and with what methods.	-	-
10. Seminars should be held for the more effective professional training of midwives on exercise during the perinatal period.	-	-
11. Swimming is the best method of exercise during pregnancy.	-	-
12. Yoga is the best method of exercise during pregnancy.	-	-
13. Pilates is the best method of exercise during pregnancy.	-	-
14. Walking is the best method of exercise during pregnancy.	-	-
15. Aerobics is the best method of exercise during pregnancy.	-	-
16. Pregnant women should perform perineal exercises.	-	-
17. Pregnant women should not exercise before 6 months of pregnancy.	-	-
18. Pregnant women should perform abdominal exercises.	-	-
19. Pregnant women should perform leg exercises.	-	-
20. The professional rights of midwives do not include training pregnant and postpartum women in exercise.	-	-
